# Toward value-based care using cost mining: cost aggregation and visualization across the entire colorectal cancer patient pathway

**DOI:** 10.1186/s12874-024-02446-5

**Published:** 2024-12-27

**Authors:** Maura Leusder, Sven Relijveld, Derya Demirtas, Jon Emery, Michelle Tew, Peter Gibbs, Jeremy Millar, Victoria White, Michael Jefford, Fanny Franchini, Maarten IJzerman

**Affiliations:** 1 Erasmus School of Health Policy and Management, Erasmus University Rotterdam, Rotterdam, the Netherlands; 2 Industrial Engineering and Business Information Systems, University of Twente, Enschede, the Netherlands; 3 University of Melbourne Centre for Cancer Research, Faculty of Medicine, Dentistry and Health Sciences, University of Melbourne, Parkville, VIC Australia; 4 Center for Healthcare Operations Improvement and Research (CHOIR), University of Twente, Enschede, The Netherlands; 5 Centre for Health Policy, Melbourne School of Public and Global Health, Faculty of Medicine, Dentistry and Health Sciences, Parkville, VIC Australia; 6 Department of Cancer Research, Peter MacCallum Cancer Centre, Melbourne, VIC Australia; 7 Cancer Research Program, School of Public Health and Preventive Medicine, Monash University, Melbourne, VIC Australia; 8 RMIT University, Melbourne, VIC Australia; 9 School of Psychology, Faculty of Health, Deakin University, Melbourne, VIC Australia; 10 Department of Medical Oncology, Peter MacCallum Cancer Centre, Melbourne, VIC Australia

**Keywords:** Costs of care, Colorectal cancer, Patient pathways, Process mining, Value-based healthcare

## Abstract

**Background:**

The aim of this study is to develop a method we call “cost mining” to unravel cost variation and identify cost drivers by modelling integrated patient pathways from primary care to the palliative care setting. This approach fills an urgent need to quantify financial strains on healthcare systems, particularly for colorectal cancer, which is the most expensive cancer in Australia, and the second most expensive cancer globally.

**Methods:**

We developed and published a customized algorithm that dynamically estimates and visualizes the mean, minimum, and total costs of care at the patient level, by aggregating activity-based healthcare system costs (e.g. DRGs) across integrated pathways. This extends traditional process mining approaches by making the resulting process maps actionable and informative and by displaying cost estimates. We demonstrate the method by constructing a unique dataset of colorectal cancer pathways in Victoria, Australia, using records of primary care, diagnosis, hospital admission and chemotherapy, medication, health system costs, and life events to create integrated colorectal cancer patient pathways from 2012 to 2020.

**Results:**

Cost mining with the algorithm enabled exploration of costly integrated pathways, i.e. drilling down in high-cost pathways to discover cost drivers, for 4246 cases covering approx. 4 million care activities. Per-patient CRC pathway costs ranged from $10,379 AUD to $41,643 AUD, and varied significantly per cancer stage such that e.g. chemotherapy costs in one cancer stage are different to the same chemotherapy regimen in a different stage. Admitted episodes were most costly, representing 93.34% or $56.6 M AUD of the total healthcare system costs covered in the sample.

**Conclusions:**

Cost mining can supplement other health economic methods by providing contextual, sequence and timing-related information depicting how patients flow through complex care pathways. This approach can also facilitate health economic studies informing decision-makers on where to target care improvement or to evaluate the consequences of new treatments or care delivery interventions. Through this study we provide an approach for hospitals and policymakers to leverage their health data infrastructure and to enable real time patient level cost mining.

**Supplementary Information:**

The online version contains supplementary material available at 10.1186/s12874-024-02446-5.

## Introduction

Recent years have witnessed significant advancements in complex care, particularly in oncology, with rapid introduction of innovative technologies and therapies. This has led to better patient outcomes but has also resulted in higher patient-specific costs due to increased complexity and specialization of care delivery [1, 2]. Recent estimates suggest that the total global economic burden of cancers will reach $25.2 trillion during the period of 2020 to 2050 [3]. This rapidly growing cost of care is unsustainable and considered one of the major challenges for health systems worldwide [2]. Value-based healthcare (VBHC) is a lens through which this issue is increasingly discussed; broadly speaking, VBHC suggests that healthcare must be organized and incentivized in a way that prioritizes outcomes and minimizes resource utilization and costs, per patient, across the integrated treatment pathway from screening or initial consultation to outcome [4]. While patient preferences and outcomes are increasingly studied, estimating costs at the patient level remains challenging [4], especially in complex care settings with extended patient journeys or repetitive treatment cycles with regular diagnostic work-ups, such as colorectal cancers (CRC). As new treatment variations and alternatives are introduced, and protocols become more tailored to individual patients, these pathways increasingly resemble interdependent webs which complicates decision-making [5–8].

Model-based health economic studies often use population-level aggregate costs and rely on ad-hoc exploration of variability or cost drivers within these aggregates, usually based on patient characteristics like age [9–12]. While suitable for evaluating interventions, this approach is less accurate for hospital-level capacity planning and process improvement [13–18]. Additionally, healthcare professionals report a lack of tools to easily identify and target specific cost drivers relevant to their local context [10, 18–20]. Determining cost drivers across patient pathways is a significant research challenge [3, 21–23], as decisions made in one treatment impact subsequent treatments' costs and outcomes, prompting calls for better tools to systematically explore variation across integrated pathways [5, 8, 18, 24–27]. Granular cost data spanning the full patient cycle, from primary care to end-of-life care, are difficult to generate [4, 28, 29], and determining variation in healthcare delivery characteristics remains a core challenge.

To address these challenges, this study presents process mining with cost estimation, which we call “cost mining,” as an approach to uncover high-cost pathways and specific cost drivers using real-world patient-level data. Process mining (PM) can complement existing health economic approaches [13, 30], by enabling patient-level cost estimates in models and generating visuals that capture patient-level variation and treatment interdependencies. PM uses low-level event data from electronic health records (EHR), such as individual consultations, procedures, and medication prescriptions, with timestamps to derive process models and discover real-world patient pathways [31]. It presents granular data in steps or phases, providing descriptive insights into patient movement through systems and resource consumption [31, 32]. As of early 2022, approximately 263 healthcare PM studies have been published [30], exploring care trajectories in acute ischemic stroke, sepsis [33], chronic diseases [34, 35], cancer [36–38], primary care [32], and COVID-19 cases [28]. This work has concluded that PM is powerful, but should include cost or resource data to make it actionable, which is what we aim to contribute in this study.

Costs have received limited attention in prior PM and VBHC studies. PM has been used to assess resource requirements and queuing improvements in emergency departments [14, 15, 18, 39], but its use in cancer care is limited due to the complexity of tracing integrated care episodes and the chronic nature of cancer [21, 22]. To support case-mix group evaluations and hospital capacity planning, additional data and analyses are needed with PM [14–16]. Cost mining can identify patient subgroups incurring additional costs due to factors like cancer stage, treatment timing, or protocol changes. It complements existing health economic methods by providing contextual information on patient pathways and the timing of treatment decisions (e.g., early-stage vs. late-stage chemotherapy). This information can serve as KPIs or benchmarks for healthcare practitioners, policymakers, and researchers, extending PM's usefulness in health services [30]. Given that only nine of 236 recently reviewed studies employed cost estimation [18, 24, 25, 27, 30], the algorithm we have developed particularly enhances PM's utility for studying the cost drivers in CRC and other complex diseases in scope for VBHC initiatives.

To develop and illustrate cost mining, we created a unique linked dataset to cover the integrated colorectal cancer (CRC) pathway in Victoria, Australia, which serves as an illustrative case study throughout the paper. Colorectal cancers, which have long trajectories beginning in primary care, are the most costly cancers in Australia [22] and the second most costly cancer globally [3], making CRC a highly relevant research context for the study of healthcare costs.

## Methods

**Fig. 1:**
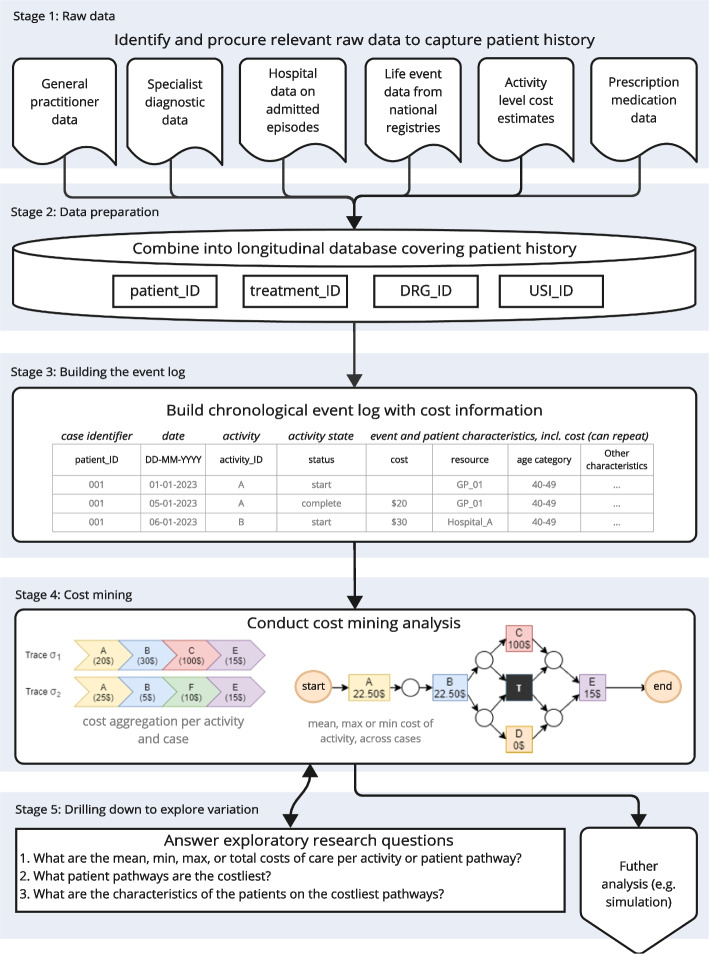
Explanatory diagram summarizing the flow of raw data into research results in the proposed method using PM with cost aggregation

In this section we describe the data requirements for cost mining integrated pathways. For a detailed description of PM techniques, we refer the reader to Munoz-Gama et al. (2023) [31] and van der Aalst (2016) [40]. In this study, we combined data from six Australian databases, detailed in appendix A and summarized in Fig. 1. The study received ethical approval by the Royal Melbourne Hospital Ethics Board through the BioGrid application (202,003/8) prior to starting.

PM structures event-level data chronologically into so called process models, which depict a linear, visualized flow of patients through a series of processes [32, 40]. Processes can have several states and attributes (e.g. a blood test can be complete or incomplete, etc.). PM describes as-is states of pathways using retrospective data; it summarizes and visualizes real world pathways, and does not make any predictions, assumptions, or imputations [29, 32, 34, 41].

### Stage 1: raw data

The method requires activity and cost information of a patient spanning the entire treatment history (screening, diagnosis, treatment, follow-up), and these activity data need to include dates or timestamps. Patients don't need to complete their treatment to be included in the analysis, as costs are estimated at the activity level, including patients still undergoing treatments is a key strength of this method. However, for group comparisons or total cost estimations, it is crucial to have treatment start dates to filter out incomplete cases and avoid downward bias in total pathway cost estimates [8]. Costs can be estimated using activity-based microcosting approaches [5, 8], or through reimbursement data such as DRGs [4, 12, 22]. The Australian reimbursements are granular, meaning that this method will produce cost statistics that capture inter-dependencies across integrated pathways. For example, the chemotherapy stage consists of several activity-based reimbursements, which means that the cost statistics will reflect differences between patients, as e.g. a patient requiring chemotherapy at a later stage of CRC may require more consultations, treatments, or regimens than a patient undergoing chemotherapy at a different CRC stage. The data requirements are summarized in the first stage of Fig. 1.

### Stage 2: data preparation

**Fig. 2:**
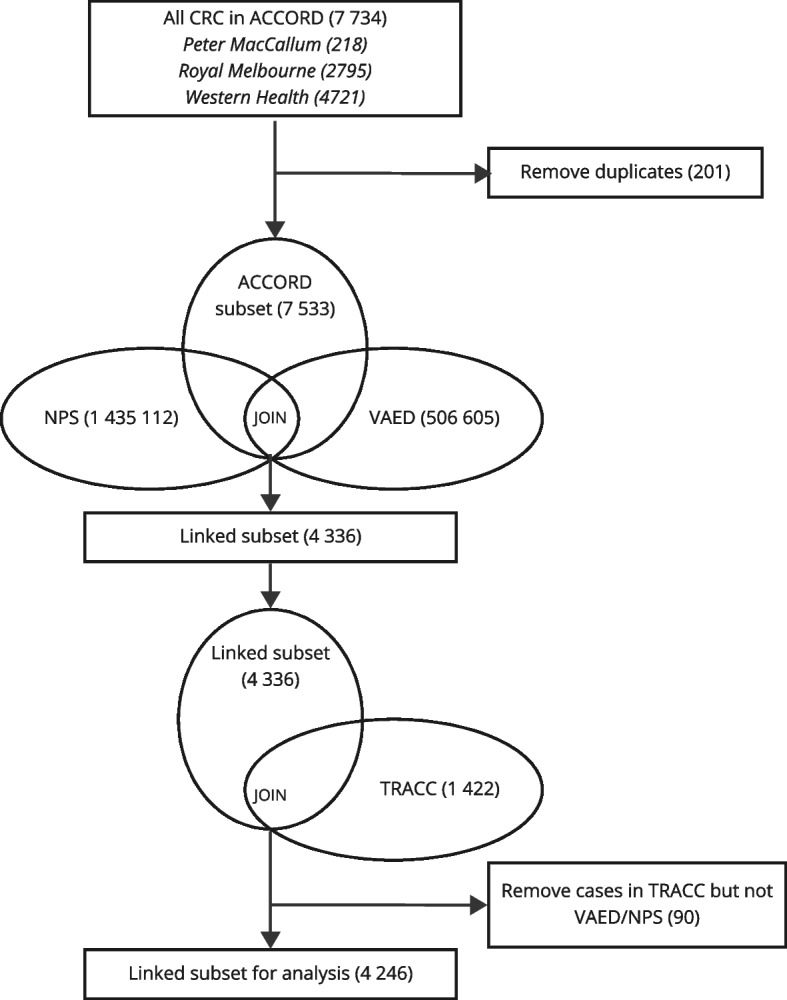
Patient record selection for the illustrative case study of colorectal cancer, resulting in a dataset of 4,246 linked unique cases with cost data at the activity or event level, covering approx. 4 million activities. For details, please refer to appendix A. Note: ACCORD: Australian Comprehensive Cancer Outcomes and Research Database; MBS: Medicare Benefits Schedule; PBS: Pharmaceutical Benefit Scheme; TRACC: Treatment of Recurrent and Advanced Colorectal Cancer; VAED: Victorian Admitted Episodes Dataset

The data need to be linked into a longitudinal database covering the integrated patient pathways and associated costs per activity. This implies that each data source identified in stage 1 of Fig. 1 needs to contain unique identifiers, e.g., anonymized patient identifiers. Further, it implies that data requirements are significant, because data linkage results in the exclusion of incomplete cases. In the CRC case shown in Fig. 2, this resulted in a set of 4246 patient records covering approximately 4 million activities (appendix A). Before conducting the analysis, it is important to assess if combining the data introduced bias through data loss, by comparing patient characteristics across data sources and the final set (appendix B).

### Stage 3: building the event log

Next, data need to be formatted in an event or activity log, which is subject to the requirements summarized in Table 1.

**Table 1:** Event log requirements, based on De Roock and Martin (2022) [30]

Element	Description
Timestamps	Dates, timestamps
Case identifier	A case identification code that is consistent and unique, e.g. one code per patient
Activity identifier	An activity identification code that is consistent and unique. This requires data cleaning and preparation to avoid cases where identical activities or events are coded inconsistently (e.g. “Chemo” vs. “Chemotherapy”)
Event status	Activity status information, e.g. started, complete, in progress associated with the timestamps
Cost of event or activity	Cost estimates, stemming from e.g., diagnosis-related group codes or microcosting
Additional data	E.g. patient characteristics, case-mix group

**Fig. 3:**
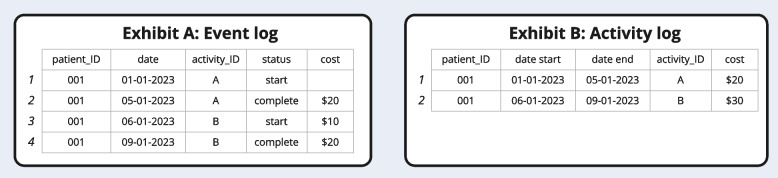
Minimum requirements of an event log or an activity log for PM with cost aggregation

An activity log contains one row per activity, with start and end times, and therefore only supports additional data at the unit of analysis of an activity as shown in Fig. 3. On the other hand, event logs offer more flexibility because they contain two or more rows per activity, as start and end points of activities are considered individual events [30, 40]. As such, it is possible to model data in which e.g. different resources are executing different elements of a single activity. A practical example of this would be a patient starting a medication-based treatment at a specialist care facility but completing it weeks later whilst being treated at a hospital for acute complications. For the purpose of cost mining, an event log is favorable to an activity log, because some healthcare activities can take weeks or months (e.g. medication treatment regimens), and others minutes (e.g. phone consultation) [30]. The largest challenge in PM in the healthcare sector is related to the inconsistent nature of the data required [30]. It can be challenging to link and combine data sources to cover integrated pathways in settings like CRC, due to the length or dispersion of treatments. Possible solutions for this include using heuristics to estimate process end times if these are unknown [8], or assuming that the start date of a specific activity signifies the end date of the prior one. In our CRC case, we did not make assumptions or imputations, because we constructed entire integrated care pathways from primary care up to outcomes like survivorship.

The event log should be built in software optimized for efficient coding, recoding, and reformatting of large data sets. We used R with the tidyverse library, which is freely available. The required event log format is shown in Fig. 3 exhibit A. Note that row 1 in the activity log contains the information from rows 1–2 in the event log. Further, note that the activity log in exhibit B loses some of the information contained in the event log (rows 3–4). The activity log cannot support data pertaining to an activity instance (start, end). Therefore, it summarizes the costs of activity B ($30) whereas the event log can show when and where these costs are incurred ($10 at start, $20 at completion).

Once the event (or activity) log is built as presented in the methods section (stage 1–3), the cost mining analysis can be conducted. Modern commercial PM software packages^1^ support the display of common statistics, such as the median number of cases per activity, but do not support customized statistics such as cost information. For this reason, we wrote a customized cost mining algorithm in Python, which is used in the following analyses (available https://github.com/chsr-uom/PM_token_decoration.)

## Results

### Stage 4: cost mining

**Fig. 4:**
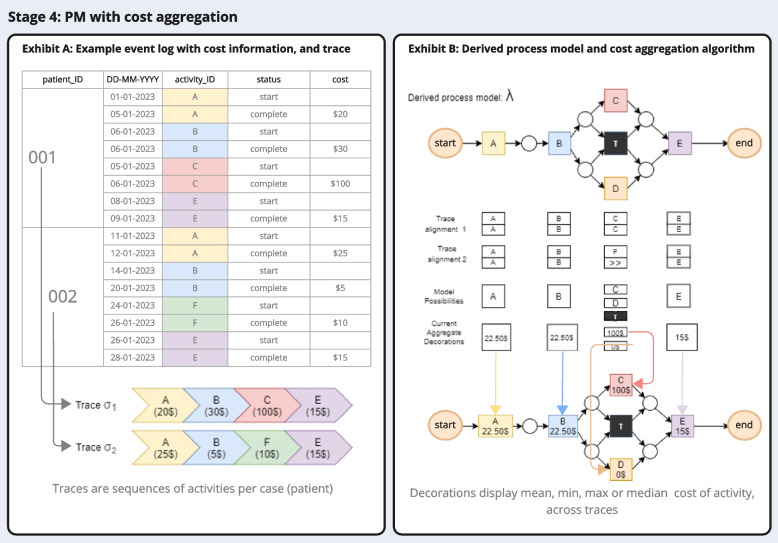
Explanatory diagram depicting how the aggregation algorithm uses the data provided in the event log (exhibit A), transforms it into traces with cost information, and then derives cost statistics by aligning traces to compute mean, median, minimum, or maximum costs (exhibit B)

The analysis starts with executing PM on the entire event log built in stage 3 using an inductive miner algorithm. It is particularly suitable to healthcare processes, because it produces inspectable process maps with a large degree simplification [32, 42–44]. Using the code we provide, the resulting process map displays cost statistics (mean, minimum, maximum, total) for each activity displayed in the form of a ‘decoration’ [45, 46], i.e. a label on the process map. For any given process model generated, the visual output provides the summary statistic of the costs per activity, based on the number of cases that have passed through the activity in that analysis. Similarly, it produces a summary statistic of the total costs of care per trace, i.e., per individual patient trajectory included. At this point, it can be useful to restrict the sample to cases that are completed to avoid under-estimating total pathway costs, by e.g. restricting the data to cases with an observed life event (e.g., survivorship, death, no treatment within 2 years). The cost mining code is described in pseudocode in appendix C. Figure 4 summarizes how the algorithm aggregates cost data; it draws on the traces derived from PM, which are sequences of events observed per case (patient) in the dataset. In simple terms, for each process map generated, the algorithm aligns all traces of the current model to calculate a statistic of the costs of each activity. In Fig. 4 exhibit B, both instances of ‘activity A’ are compared and translated into a mean (in this case, the average of $20 and $25 is $22.50). To do so, the algorithm accounts for all patients that have undergone activity A, across all traces (sequences of activities). Because, for example, only a single instance of activity C is observed in this hypothetical example, the label returns the value of $100 attached to activity C. In a final step, the code attaches the generated statistic value to the process map as a ‘decoration’ label [45, 46].

### Stage 5: drilling down to explore variation

The generated process model will display pathways, which warrant further exploration in terms of e.g. case-mix groups, diagnoses, or indications, which we term ‘drilling down’ into the data to further understand rare, desirable, or undesirable pathways and cost drivers [30, 32, 40]. This allows us to quantify mean and range per patient group as well as to determine subgroups based on certain cost outcomes (e.g. most expensive).

**Fig. 5:**
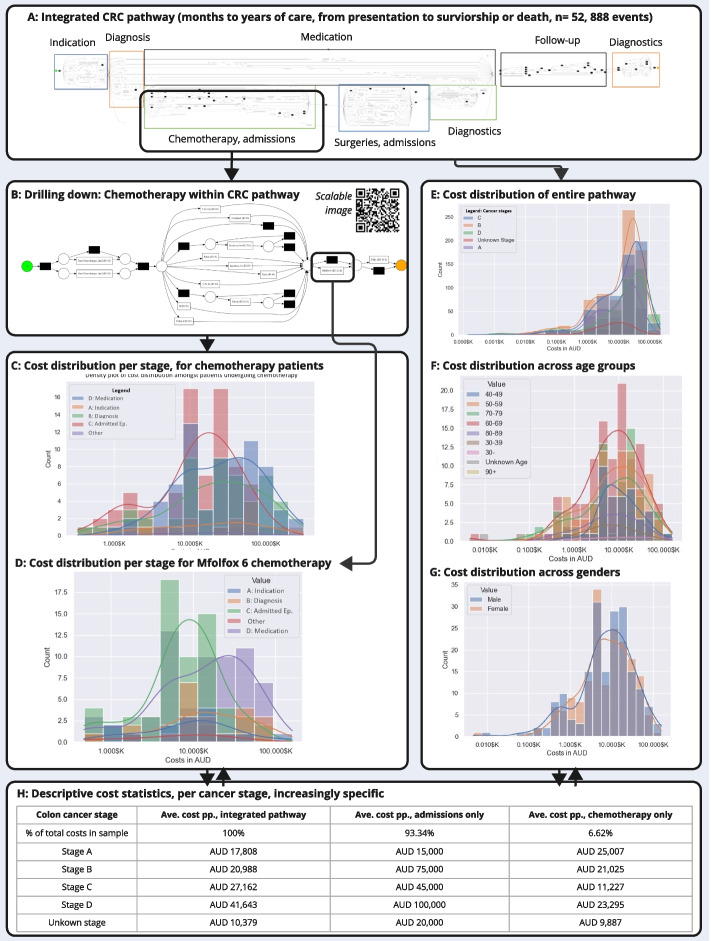
Illustrative results gained from PM with cost aggregation for CRC pathway, with particular focus on chemotherapy, to show how the method supports ‘drilling down’ to understand where high costs are being incurred, for which patient groups, and which treatment modalities

We illustrate the method in Fig. 5 using the CRC case. We were able to identify crucial decision points (after which pathways were significantly different in complexity and costs), pinpoint costly processes, and make case-mix comparisons across groups (sex, age group, tumour location, tumour stage, CRC-type, patient’s rurality, and indigenous status; see right side of Fig. 5). In CRC, we found that the average costs of care ranged from $10,379 AUD to $41,643 AUD per patient (Fig. 5 panel H) and differed significantly per stage of treatment.

Drilling down in our data revealed that colon cancer was associated with significantly greater costs across the entire care continuum than rectal cancer, and admissions and chemotherapy were by far the most expensive elements of treatment (Fig. 5, panels C, D). Admitted episodes (*n* = 1,965 patients) cost a total of $56.6 M AUD (93.34% of total costs covered by the data, $ 60,63 M AUD). In comparison, the total cost of chemotherapy drug treatments (*n* = 218 patients) was 6.62% of total costs. GP visits, diagnostic testing, and prescriptions made up less than 0.01% of the total costs. Our results reveal that treatment-related factors, namely cancer stage, significantly related to costs (Fig. 5, panel H).

When drilling down into the chemotherapy treatments, treatment with a specific regimen (Mfolfox 6; Fig. 5 panel D) was extremely costly, at an average cost of $35 K AUD per patient. However, these costs significantly varied across the different cancer stages, with stage C cancer patients incurring much higher costs associated with the Mfolfox 6 chemotherapy regimen than other patients, which warrants future qualitative and quantitative research. In this way, this exploratory technique can account for the temporal nature of care, as the costs of e.g. receiving chemotherapy during late-stage cancer are higher than early-stage. In future, if protocol changes are introduced to e.g. circumvent the use of Mfolfox 6 during stage C CRC, the cost and duration impact of this change can be traced using cost mining.

## Discussion

In this methodological paper, we draw on recent PM work in healthcare settings [13, 18, 25, 31, 41, 46] to develop and trial a method to support VBHC. Because cost mining aggregates cost information across entire patient journeys using real life data, this method translates large volumes of data into useful and practical information with which care can be made more efficient, accessible, and sustainable. In doing so, we have answered several recent calls for research [47–50] and built on recent methodological work calling for PM with financial KPIs [30].

### Applications for cost mining

This method is relevant to achieving process efficiency, cost reduction, improved resource allocation, continuous process improvement, and data driven medical decision-making to ensure financial sustainability in a landscape of increasing complexity.

At the international level, this method could facilitate financial benchmarking across different standards of care and healthcare systems by comparing large patient cohorts in terms of patient pathways, to identify high-cost or long-duration pathways to target with interventions. Thus, it would supplement ongoing analyses, or large retrospective or prospective cohort studies, by providing patient flow information alongside common health economic analyses [50].

At the national level, this method can aid researchers and policy-makers in tracing and evaluating increasing healthcare delivery variation, for instance in response to medical protocol changes over time, technological advancements in medicine, and digitalization of healthcare service delivery. This is particularly relevant in countries that feature strong or increasing care concentration, such as the Netherlands [51]. Further, cost mining could uncover the long-term consequences of shifting standards of care, by mapping and aggregating the costs associated with specific procedural guidelines by comparing patient groups before and after policy changes, or across locations. Even in less fragmented systems (e.g., US) where patient-level data is more integrated, cost mining still holds relevance. Although one could directly determine costs from patient-level data, cost mining offers the ability to uncover underlying patterns, sequences, and relationships within the care process, which can complement traditional microcosting studies by providing contextual information, and by exploring how sequences or timing impact costs, outcomes, and durations.

At the clinical level, it can reveal whether specific patient groups are consuming disproportionately more care than others, as we have demonstrated in our CRC case, or face significantly longer or more invasive trajectories. This may also enable assessment of care equity by, for example, comparing advantaged to disadvantaged or underrepresented patient groups. By exploring utilization patterns in a systematic way using cost mining, future research could identify whether disadvantaged groups are consuming more or less care than their counterparts, which opens up new avenues for prevention and intervention strategies relating to health equity. Moreover, this information would, in turn, provide valuable insights for future health technology assessments or cost-effectiveness assessments, enabling them to estimate the process and cost impact of e-health technologies from financial, sustainability, and equity perspectives [52]. Further, this method could be used to explore the economic impact of prevention, early diagnosis [21, 22, 53] and excessive routine diagnostics [54] or prescriptions [55] by assessing and comparing integrated pathways longitudinally.

### Costs of CRC in Australia

The contribution of the present study is that we find that cancer stages relate to costs, and that costs of specific elements of CRC care are dependent on the relative timing in which they are administered during a patient’s integrated pathway. Previous studies in New Zealand [56], England [57], the US [58], Europe [59], and Australia [21, 22], reported on costs of care for CRC cases in relation to control variables like age and sex. Building on this, we report treatment-specific factors like cancer stage as explanatory factors of cost variation. Only two prior studies found CRC costs to relate to cancer stage [22, 57]. Our results extend these findings by showing that stages B and C have the highest total costs, and stages C and D have the highest mean cost per patient, which suggests that treatment-related factors and timing influence costs. Whilst prior work focused on treatments [21, 58], we included primary care and life events and captured the integrated pathway, covering all treatments and events related to CRC. Importantly, our results show that chemotherapy costs depend on the cancer stage, with specific patient groups requiring high-cost regimens like Mfolfox 6 at specific stages (e.g., stage C) relating to high per-patient costs. These findings extend recent work and illustrate the benefits of mapping integrated patient pathways with data from multiple providers (e.g., GPs) to explore costs in relation to cancer stage and timing of treatments. By incorporating the entire pathway, we show that the total healthcare burden of CRC in Australia is predominantly related to inpatient episodes, but that per-patient costs within chemotherapy vary and relate to specific regimes in specific cancer stages. Future research should utilize cost mining to investigate whether preventative interventions or earlier screening and diagnosis lead to quicker patient pathways or comparatively lower-cost inpatient and chemotherapy episodes, given the significant correlation between cancer stage at the time of treatment and costs. Beyond CRC, future studies could expand on our algorithm to develop routine cost mining evaluations in other costly contexts, complementing and informing traditional economic and qualitative methods.

### Limitations of cost mining

Cost mining has limitations inherent to PM and the use of historical patient data, namely significant data requirements, descriptive nature, and a lack of predictive power. The method primarily visualizes as-is states using retrospective data, describing costs faced by patients who have completed (parts of) their care trajectory. This may not reflect current costs for treatments with recent technological developments, and the analysis should be repeated periodically to discover new pathways as they occur.

Due to the descriptive nature of this analysis, the method requires significant volumes of data to be representative, and results must be interpreted cautiously. The method can uncover high-cost pathways and identify paths or patient groups that completed unusually costly pathways. However, the method cannot be used to judge whether medical decisions were cost-effective not, and the user must assume that pathways were chosen out of medical necessity. The resulting visualizations should therefore be used to uncover cost drivers to inform VBHC projects, or to identify patient groups that face unusually costly or lengthy treatments, and should be used in tandem with methods like micro costing or cost-effectiveness analyses [8], and qualitative approaches like realist evaluations that uncover situational or causal mechanisms [55]. Low patient numbers in specific branches of pathways are not problematic if the patient number is representative of the entire study population. Because the analysis is descriptive, it is sensitive to omissions, so excluded cost or activity data will result in an underestimation of cost statistics. Lastly, some contexts may be difficult to model with PM. Systems with free choice of GP and healthcare provider are challenging due to fragmented patient data across providers, necessitating manual linkage. In contrast, systems with seamless electronic health records, like those in the Netherlands, are easier to model as they capture all general and specialist care regardless of location.

## Conclusion and future research

The cost mining method identified inpatient and chemotherapy episodes as particularly costly in Australian CRC care, driven by cancer stage, accounting for 99% of the $60.63 M AUD economic burden on the Australian health system (2012–2020). Our analysis underscores the benefits of linked registries and cost mining for assessing healthcare costs across integrated pathways to inform VBHC projects. Future research could extend this method, and address some of its limitations, using predictive PM utilizing machine learning [60], to produce process maps that are not only actionable but also predictive. Additionally, our method relies on static cost estimates per activity using DRG data, whereas future work could develop algorithms that allow resource usage to vary per activity per patient, using cost equations [8].

## Supplementary Information

Supplementary Material 1.

## Data Availability

The linked dataset that was analyzed and used for this study is available from BioGrid (https://www.biogrid.org.au) on a secured server subject to ethics approval. The data is not publicly available, to preserve privacy and anonymity.

## Footnotes

1 https://www.fluxicon.com/disco (commercial). https://www.celonis.com (commercial). https://www.apromore.org (commercial). https://www.promtools.org (free). https://pm4py.org (free for use in Python). https://www.bupar.net (free library for use in R).
