# Supplementary material for: Toward value-based care using cost mining: cost aggregation and visualization across the entire colorectal cancer patient pathway
Source: BMC Med Res Methodol. 2024 Dec 27;24:321. doi: 10.1186/s12874-024-02446-5 (PMC11681630; doi:10.1186/s12874-024-02446-5)
Supplement: Supplementary file 1 — Supplementary Material 1. [file 12874_2024_2446_MOESM1_ESM.docx]

# Appendix A: Data linkage in the case of colorectal cancer in Victoria, Australia

To build the longitudinal database described in **figure 1** of the manuscript for CRC in Australia, we integrated data from multiple sources, covering three major hospitals in Melbourne. Data from two clinical registries, ACCORD^[[1]](#footnote-1)^ and TRACC^[[2]](#footnote-2)^, were linked with the three hospital’s administrative datasets (VAED^[[3]](#footnote-3)^) and primary care data (NPS Medicine Insight). The merged data provided a comprehensive view of patient encounters, treatments, and medication usage related to colorectal cancer. Activity costs were derived from VAED (WIES factor^[[4]](#footnote-4)^) and NPS Medicine Insight, through cost retrieval of medical services and pharmaceutical prescription item numbers respectively available on MBS^[[5]](#footnote-5)^ and PBS^[[6]](#footnote-6)^ websites. The project was part of a larger multi-center research program and had received ethics approval by Royal Melbourne Hospital Ethics Board through the BioGrid application (202003/8).

ACCORD is a comprehensive cancer outcomes and research database that collects information on patients with various tumor types. For this study, patients diagnosed with colorectal cancer were selected. ACCORD contains patients' clinical characteristics, such as tumor type and treatments received, and utilizes an encrypted unique swap identifier (USI) for data linkage. The Victorian Admitted Episodes Dataset (VAED) provides information on hospital admissions, diagnoses, and procedures in Victorian hospitals. The dataset includes encounters with the healthcare system and covers patients admitted to public or private hospitals, extended care facilities, or day procedure centers. The NPS Medicines Insight database consists of de-identified electronic health records from Australian general practices. It includes information on patient encounters, investigations, and prescribed medications. After linking the ACCORD subset from the three hospitals with the NPS MedicineInsights and VAED data, 4336 unique patient records remained.

TRACC focuses on the treatment of recurrent and advanced colorectal cancer, enrolling patients from Australian and Hong Kong hospitals. After linking the TRACC data to the ACCORD subset, the linked dataset contained 4246 unique patient records.

In sum, we linked data from ACCORD, TRACC, VAED, and NPS Medicines Insight to build a longitudinal database of all patient encounters (activities, consults, surgeries, etc.) and medication usage related to CRC. Activity costs were derived from VAED (WIES factor) and NPS MedicineInsights. In NPS MedicineInsights, information related to prescriptions and medical services provided to patients by general practitioners is available. This information corresponds to item numbers that are found in the Pharmaceutical Benefits Scheme (PBS, drugs) and Medicare Benefit Schedule (MBS, medical services). In the linked dataset, there is no cost data available within NPS MedicineInsights, however, it is possible to retrieve individual item number cost online on PBS and MBS websites. Therefore, both websites were scraped to retrieve cost data of all available items and data was uploaded onto the secure server to attribute cost to items found in NPS MedicinesInsight.

In the case of CRC in Australia, we linked five sources of data to meet the requirements discussed above. These data sources are summarized in **appendix A** **table 1**, per phase of treatment relevant to CRC, and together these data sources cover the data requirements described in the main manuscript. Each row details the data sources, depicted in **appendix A** **figure 1**. In case of CRC, patients pass through 4 stages of care. Lastly, life event data is required to define an end state to each patient pathway (e.g., survivorship). To be able to link, merge and use such data, ethical approval from the relevant institution(s) should be requested, detailing how the data will be/is anonymized to ensure privacy [1–3]. It is considered best practice to categorize data that is not needed in direct form, such as age or BMI, which in combination with each other could enable the identification of individuals that fall outside the normal distribution [1]. The combined registry data captures a total economic burden of $ 60,63M AUD, across approximately 4000K/4 million care activities delivered. A noticeable shortcoming of this dataset is that it covers very few patients treated with radiation therapy (about 30) whereas it would be expected that about 15% of patients were treated with radiation [4]. This may have been caused by the data joining process, whereby several patients were excluded if data was incomplete following the merge this or this may be related to inconsistent reimbursement (DRG) coding.

| General requirements for process mining with cost aggregation | | | Illustrative CRC case data sources |
| --- | --- | --- | --- |
| Pathway | Data | Timestamp data (examples) | Sources |
| (A) Indication | General practitioner data | - GP visit - Screening referral - Detected in another admission | NPS  MedicineInsight, |
| (B) Diagnosis | Specialist diagnostics data | - Imaging techniques (CT/MRI) - Colonoscopy - Histology | NPS  MedicineInsight, |
| (C) Admitted Episodes | Hospital data on admitted episodes | - Surgeries - Admission for chemotherapy - Palliative care | VAED,  WIES Factor,  CPI |
| (D) Medications | Prescription medication data | - Chemotherapy drug during admission - Drug prescribed for side effect | NPS MedicineInsight, ACCORD |
| €  Life events | Life event data from national registries | - Diagnosis - Death, survivorship - Lost to follow-up | TRACC, ACCORD |
| All | Cost data | - Annual standardized service costs - DRGs, DBCs, or reference prices - Cost estimates derived from activity-based costing techniques | NPS MedicineInsight, VAED (WIES Factor) |

**Appendix A Table 1.** Type of activities in a phase of a process within Australian CRC context. Note: VAED: Victorian Admitted Episodes Dataset; ACCORD: Australian Comprehensive Cancer Outcomes and Research Database; TRACC: Treatment of Recurrent and Advanced Colorectal Cancer; PBS: Pharmaceutical Benefit Scheme, MBS: Medicare Benefits Schedule, CPI: Consumer price index


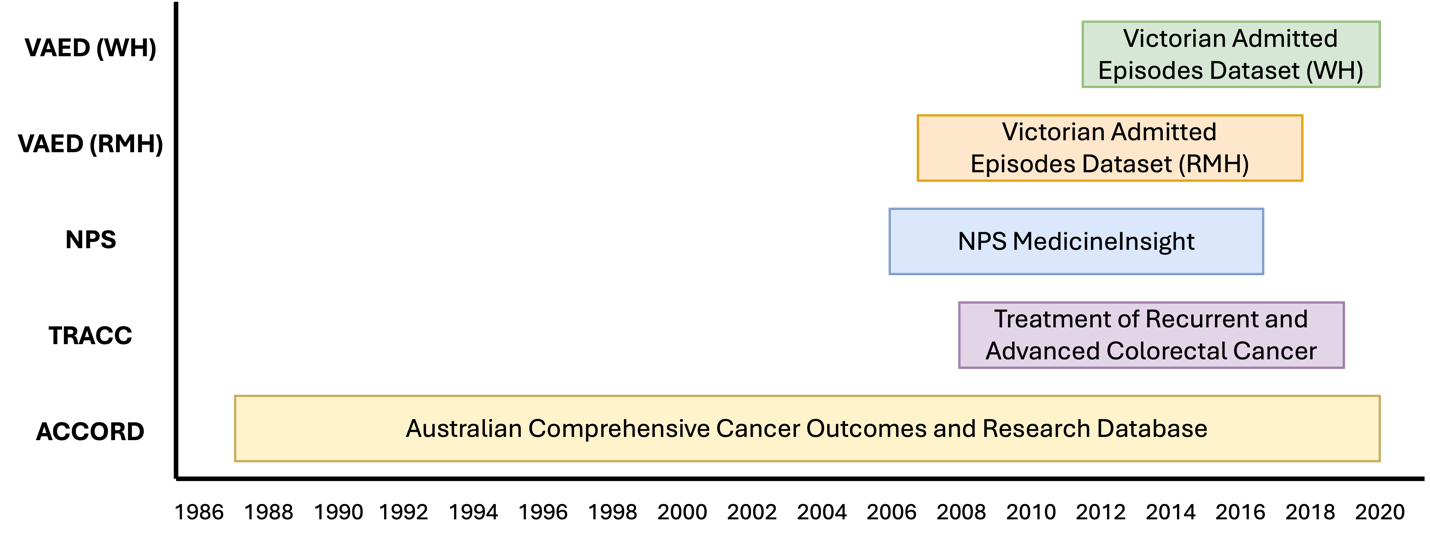


**Appendix A Figure 1:** Summary of data sources used for CRC pathway in Australia and the timeframe they cover.

**Appendix A references**

1 Xu L, Jiang C, Chen Y, Wang J, Ren Y. A framework for categorizing and applying privacy-preservation techniques in big data mining. Computer. 2016 Feb 11;49(2):54-62.

2 Pika A, Wynn MT, Budiono S, Ter Hofstede AH, van der Aalst WM, Reijers HA. Privacy-preserving process mining in healthcare. International journal of environmental research and public health. 2020 Mar;17(5):1612.

3 Elkoumy G, Fahrenkrog-Petersen SA, Sani MF, Koschmider A, Mannhardt F, Von Voigt SN, Rafiei M, Waldthausen LV. Privacy and confidentiality in process mining: Threats and research challenges. ACM Transactions on Management Information System (TMIS). 2021 Oct 5;13(1):1-7.

4 Mackenzie, P., Vajdic, C., Delaney, G., Comans, T., Agar, M., Gabriel, G., & Barton, M. (2022). Development of an Age- and Comorbidity- Adjusted Optimal Radiotherapy Utilisation Rate for Patients with Lung Cancer. *Journal of Geriatric Oncology*, *13*(8), S4–S5. https://doi.org/10.1016/S1879-4068(22)00255-7

# Appendix B: Patient characteristics per dataset constructed

|  | Life events, from ACCORD (N=4246) | Admitted episodes, from VAED (N=3233) | Chemo. Episodes from VAED  (N=461) | Diagnostic test  (N=50) | GP visits, from NPS  (N=163) | Prescriptions from NPS and ACCORD (N=84) |
| --- | --- | --- | --- | --- | --- | --- |
| **Gender** |  |  |  |  |  |  |
| F | 1792 (42.2%) | 1357 (42.0%) | 175 (48.9%) | 24 (48.0%) | 74 (45.4%) | 43 (51.2%) |
| M | 2454 (57.8%) | 1876 (58.0%) | 183 (51.1%) | 26 (52.0%) | 89 (54.6%) | 41 (48.8%) |
| **Age Group** |  |  |  |  |  |  |
| <30 | 42 (1.0%) | 38 (1.2%) | 7 (2.0%) | 2 (4.0%) | 2 (1.2%) | 2 (2.4%) |
| 30-39 | 113 (2.7%) | 84 (2.6%) | 16 (4.5%) | 1 (2.0%) | 6 (3.7%) | 2 (2.4%) |
| 40-49 | 311 (7.3%) | 247 (7.6%) | 40 (11.2%) | 4 (8.0%) | 18 (11.0%) | 7 (8.3%) |
| 50-59 | 698 (16.4%) | 522 (16.1%) | 82 (22.9%) | 8 (16.0%) | 29 (17.8%) | 9 (10.7%) |
| 60-69 | 1220 (28.7%) | 950 (29.4%) | 110 (30.7%) | 16 (32.0%) | 55 (33.7%) | 32 (38.1%) |
| 70-79 | 1181 (27.8%) | 902 (27.9%) | 70 (19.6%) | 11 (22.0%) | 30 (18.4%) | 19 (22.6%) |
| 80-89 | 572 (13.5%) | 416 (12.9%) | 29 (8.1%) | 8 (16.0%) | 20 (12.3%) | 10 (11.9%) |
| 90+ | 37 (0.9%) | 27 (0.8%) | 1 (0.3%) | 0 (0.0%) | 1 (0.6%) | 2 (2.4%) |
| Unknown | 72 (1.7%) | 47 (1.5%) | 3 (0.8%) | 0 (0.0%) | 2 (1.2%) | 1 (1.2%) |
| **Tumour location** |  |  |  |  |  |  |
| Colon | 2580 (60.8%) | 1983 (61.3%) | 218 (60.9%) | 31 (62.0%) | 95 (58.3%) | 52 (61.9%) |
| Rectal | 1508 (35.5%) | 1153 (35.7%) | 132 (36.9%) | 19 (38.0%) | 64 (39.3%) | 30 (35.7%) |
| Other | 19 (0.4%) | 17 (0.5%) | 2 (0.6%) | 0 (0.0%) | 0 (0.0%) | 0 (0.0%) |
| Undefined | 139 (3.3%) | 80 (2.5%) | 6 (1.7%) | 0 (0.0%) | 4 (2.5%) | 2 (2.4%) |
| **Tumour Stage** |  |  |  |  |  |  |
| A | 763 (18.0%) | 591 (18.3%) | 37 (10.3%) | 10 (20.0%) | 37 (22.7%) | 17 (20.2%) |
| B | 1250 (29.4%) | 923 (28.5%) | 77 (21.5%) | 17 (34.0%) | 43 (26.4%) | 18 (21.4%) |
| C | 1037 (24.4%) | 802 (24.8%) | 111 (31.0%) | 10 (20.0%) | 30 (18.4%) | 19 (22.6%) |
| D | 646 (15.2%) | 526 (16.3%) | 106 (29.6%) | 9 (18.0%) | 34 (20.9%) | 25 (29.8%) |
| Unknown | 550 (13.0%) | 391 (12.1%) | 27 (7.5%) | 4 (8.0%) | 19 (11.7%) | 5 (6.0%) |
| **Etnicity/ Indigenous Status** |  |  |  |  |  |  |
| Aboriginal | 507 (12.0%) | 297 (9.2%) | 18 (3.9%) | 10 (20.0%) | 18 (11.0%) | 9 (10.7%) |
| Not Ab/TS | 3462 (81.6%) | 2824 (87.4%) | 335 (72.7%) | 36 (72.0%) | 135 (82.8%) | 70 (83.3%) |
| Torres Strait | 18 (0.4%) | 18 (0.6%) | 1 (0.2%) | 0 (0.0%) | 0 (0.0%) | 0 (0.0%) |
| Unknown | 254 (6.0%) | 93 (2.9%) | 107 (23.2%) | 4 (8.0%) | 10 (6.1%) | 5 (6.0%) |
| **Remoteness** |  |  |  |  |  |  |
| Inner Regional | 224 (5.3%) | 151 (4.7%) | 18 (5.0%) | 0 (0.0%) | 0 (0.0%) | 1 (1.2%) |
| Major City | 3959 (93.2%) | 3056 (94.5%) | 338 (94.4%) | 48 (96.0%) | 160 (98.2%) | 82 (97.6%) |
| Outer Regional | 36 (0.8%) | 18 (0.6%) | 0 (0.0%) | 0 (0.0%) | 1 (0.6%) | 0 (0.0%) |
| Remote | 1 (0.0%) | 0 (0.0%) | 0 (0.0%) | 0 (0.0%) | 0 (0.0%) | 0 (0.0%) |
| Unknown | 26 (0.6%) | 8 (0.2%) | 2 (0.6%) | 2 (4.0%) | 2 (1.2%) | 1 (1.2%) |

**Appendix B table 1:** Patient characteristics per data source or registry used.

# Appendix C: Cost aggregation algorithm

The objective of the algorithm is to enhance the process maps with additional information regarding the costs of each of the executed process steps. Thus, the extension can aggregate the value of a custom defined numeric value, over all aligned traces to a petri net. This function takes in four objects:

1. an event log 𝑳_h_ which contains a set of traces to be aligned to the petri net,

2. the discovered petri net itself,

3. The start- and end markings M_0_ and M_f_

4. an aggregation function, such as the median, the mean, the sum.

The algorithm pseudocode is provided in **appendix C figure 2**. The initialization step of the algorithm initializes an empty list **O** of numeric values, where the current cost value will be stored (step 1). Then first, from the event log 𝑳_h_, the set of traces 𝛷_h_ is stored and a list of all the activities or transition Τ_H,i_ in the model λ_H_. Secondly, For each level of hierarchy evaluated (**H**), each trace σ in 𝛷_h_ is aligned to model λ_H_ (step 2). Then, for each activity 𝑎𝑗(𝑖) in the trace σ aligned to transition T_H,i_ , the associated cost value is aggregated by the specified aggregation function (in this case mean, although minimum, maximum, or median are also possible) and stored in the initialized list **O** (step 3). When all traces have been completed, the list **O** is concatenated to the list of transitions T_H,I_ in the model λ_H_, resulting in an annotated model (step 4). This results in a decorated petri net with aggregated costs added to each transition or each activity in the petri net. The costs are then added as an additional attribute, summing all costs of the activities for the included cases (output).

**Appendix C Figure 2.** Pseudocode node aggregation in petri net.

1. ACCORD; Australian Comprehensive Cancer Outcomes and Research Database [↑](#footnote-ref-1)
2. TRACC; Treatment of Recurrent and Advanced Colorectal Cancer [↑](#footnote-ref-2)
3. VAED; Victorian Administrative Episodes Data hospital administrative Datasets [↑](#footnote-ref-3)
4. WIES; Weighted Inlier Equivalent Separation factor [↑](#footnote-ref-4)
5. MBS; Medicare Benefits Schedule [↑](#footnote-ref-5)
6. PBS; Pharmaceutical Benefit Scheme [↑](#footnote-ref-6)
